# Editorial: Crucial Role of Redox Signaling in the Regulation of Heart Health

**DOI:** 10.2174/157340310793566082

**Published:** 2010-11

**Authors:** Dipak K Das

Overwhelming evidence exists in the literature to support the role of reactive oxygen species (ROS) as intracellular signaling molecules. Several degenerative diseases including coronary heart disease, have been linked with the overproduction of ROS. Many coronary heart diseases including ischemic heart disease cause cardiomyocytes to face conditions that shift their redox status to undergo a drastic change subjecting them to oxidative stress. Cardiomyocytes contain enzymes that constantly generate ROS and intracellular redox buffer in response to a specific stress developed from the dsease process. Depending on the amount of antioxidant reserve, ROS is either destroyed or persist. If ROS is overwhelming, they destroy cells, but on the other hand, if ROS activity if minimal, they may function as signaling molecules and function as savior rather than destroyer. The intention of this minireview series is to provide evidence that ROS indeed function as signaling molecules and serve to protect the cardiac cells against the disease. 

The review by Madhu Anand Srivastava, Professor of Physiology at the University of Montreal, Canada, entitled *Modulation of Gi proteins in Hypertension: Role of Angiotensin II and Oxidative stress* is an excellent example how hypertension is controlled by ROS and redox signalling. The enhanced or unaltered levels of inhibitory guanine nucleotide regulatory troteins (G-proteins - Giα-2 and Giα-3) and mRNA have been reported in different models of hypertension, whereas Gsα levels were shown to be unaltered. These changes in G-protein expression were associated with Gi functions. The enhanced levels of Giα proteins precede the development of blood pressure and suggest that overexpression of Gi proteins may be one of the contributing factors for the pathogenesis of hypertension. Enhanced oxidative stress in hypertension due to Angiotensin II is responsible for the enhanced expression of Giα proteins observed in hypertension. The review discusses the mechanism by which oxidative stress enhances the expression of Gi proteins through the activation of mitogen activated protein (MAP) kinase activity.

The next article by Professor Lindsay Brown from the University of Queensland, Brisbane, Australia describes how a redox sensitive polyphenolic compound resveratrol *improves cardiova-scular function in DOCA-salt hypertensive rats. *This study has determined whether treatment with resveratrol prevented cardiac fibrosis and the decreased cardiovascular function in the DOCA-salt hypertensive rat as a model of human hypertension. In these DOCA-salt rats, resveratrol decreased inflammatory cell infiltration, decreased cardiac fibrosis (left ventricular interstitial and perivascular collagen content) and improved cardiac and vascular function. Resveratrol attenuated other features of cardiovascular remodelling such as increases in systolic blood pressure, left ventricular wet weight, left ventricular wall thickness, diastolic stiffness constant, as well as decreased cardiac contractility and prolonged action potential duration characteristic of DOCA-salt rats. In summary, the redox-sensitive compound resveratrol, at a nutritionally relevant dose, prevents or attenuates the adverse changes in the cardiovascular system. We propose that the anti-inflammatory and anti-fibrotic effects of resveratrol are responsible, at least in part, for its amelioration in cardiovascular remodelling in DOCA-salt rats. 

Next, Professor Shyamal Goswami and Dipak K Das from the University of Connecticut School of Medicine, Farmington, Connecticut, USA describes the role of HIF-1a and oxygen sensing in cardiac ischemia. Although our knowledge of the biochemistry and physiology of oxygen transport is century old, recent development of sophisticated tools of biophysical chemistry revealed that tissue oxygenation and oxygen sensing is a highly evolved process, especially in mammals. Perturbation of normal oxygen supply is associated with diseases like tumorigenesis, myocardial infarction and stroke. Available information suggests that when tissue oxygen supply is limited, mitochondria emanate signals involving reactive oxygen species generation which in turn stabilizes oxygen sensing transcription factor HIF-1. Upon stabilization, HIF-1 elicits necessary genetic response to cope with the diminished oxygen level. In view of such critical role of HIF-1 in cellular oxygen sensing, recently there has been a heightened interest in understanding the biology of HIF-1 in the context of cardiovascular system. 

Naranjan S. Dhalla, distinguished professor of the university of Manitoba, Winnipeg, Canada described how state-of-the-art technique of cardioprotection leads to subcellular remodelling through redox signaling. Cardiac function is compromised by oxidative stress which occurs upon exposing the heart to ischemia reperfusion (I/R) for a prolonged period. The reactive oxygen species (ROS) that are generated during I/R incur extensive damage to the myocardium and result in subcellular organelle remodeling. The cardiac nucleus, glycocalyx, myofilaments, sarcoplasmic reticulum, sarcolemma, and mitochondria are affected by ROS during I/R injury. On the other hand, brief periods of ischemia followed by reperfusion, or ischemic preconditioning (IPC), has been shown to be cardioprotective against oxidative stress by attenuating the cellular damage and alterations of subcellular organelles caused by subsequent I/R injury also occur in IPC. Endogenous defense mechanisms, such as antioxidant enzymes and heat shock proteins, are activated by IPC and thus prevent damage caused by oxidative stress. Although these cardioprotective effects of IPC against I/R injury are considered to be a consequence of changes in the redox state of cardiomyocytes, IPC is considered to promote the production of NO which may protect subcellular organelles from the deleterious actions of oxidative stress. The actions of various endogenous cardioprotective interventions are discussed to illustrate that changes in the redox state due to IPC are cardioprotective against I/R injury to the heart. 

In a highly original paper, Professor Hannah Vasanthi and Rajamanickam from the Advanced Research in Indian System of Medicine, SASTRA University, and Sri Ramachandra Medical College and Research Institute, Sri Ramachandra University, Tamilnadu, India, describe how *a flavonoid rich fraction (FRF) of Dioscorea bulbifera Linn. (Yam) enhances mitochondrial enzymes and antioxidant status and thereby protects heart from isoproterenol induced myocardial infarction. *FRF when intervened for a period of 35 days prior to isoproterenol (ISO) challenge to rats, alterations in the antioxidant status in the mitochondria were recognized in the heart tissue of ISO induced rats. ISO induced rats pretreated with FRF ameliorated the lipid peroxidation and thereby enhanced the antioxidant status as evidenced by the increase in the reduced glutathione (GSH) content and the activity of antioxidant enzymes. Moreover, the tricarboxylic acid cycle enzymes such isocitrate dehydrogenase (ICDH), succinate dehydrogenase (SDH), malate dehydrogenase (MDH) and α-ketoglutarate dehydrogenase (α-KGDH), which were found decreased in the ISO induced rats showed an enhanced activity in FRF pretreated rats. The activity of NADH dehydrogenase and cytochrome-C-oxidase the enzymes, which transfer the electron in the electron transport chain (ETC) was also increased significantly (*p*<0.05) in FRF (150 mg/kg) pretreated rats, when compared with ISO induced rats. These results suggest the cardioprotective effect of FRF of *Dioscorea bulbifera* Linn. in ISO induced MI by attenuating the lipid peroxidation by scavenging free radicals and modulating the energy producing mitochondrial enzymes.

Professor Nesrin Kartal Ozer from the Marmara University,Haydarpasa, Turkey, describes role of lipid rafts and redox signaling in cholesterol induced atherosclerosis.

Kenichi Watanabe, M.D., Ph.D. professor of Clinical Pharmacology at the Nigata University, Nigata, Japan, described the role of different signaling pathways and oxidative stress in diabetic cardiomyopathy. 

Finally, Professor Asok Srivastava from the Department of Medicine, University of Montreal, Qubec, Canada, in a highly original paper, describes how a potent vasoconstrictor, endothelin-1, induces redox signaling through nitric oxide/cGMP system in vascular smooth muscle cells.

These reviews and original papers demonstrate that low physiologically relevant concentration of ROS can regulate a variety of key molecular mechanisms. Cardiovascular diseases cause redox changes in various heart cells. The molecular implications of such change are not fully characterized. The eight component articles of this minireview series discuss various aspects of cardiac diseases regulated by redox signaling.

